# Short overview on the current standard of treatment in newly diagnosed multiple myeloma

**DOI:** 10.1007/s12254-018-0383-3

**Published:** 2018-02-21

**Authors:** Ella Willenbacher, Agnes Balog, Wolfgang Willenbacher

**Affiliations:** 1 Internal Medicine V, Hematology & Oncology, Innsbruck Medical University, Anichstraße 35, 6020 Innsbruck, Austria; 2 Oncotyrol, center for personalized cancer medicine, Innsbruck, Austria; 3 Blood Transfusion Center, Innsbruck, Austria

**Keywords:** Multiple myeloma, Multiple myeloma treatment indication, Multiple myeloma 1st line therapy, Multiple myeloma transplant eligible, Multiple myeloam transplant ineligible

## Abstract

The treatment of newly diagnosed multiple myeloma has changed dramatically over the past 20 years, from near uniform application of chemotherapy to a patient performance status- and risk-based approach. Furthermore, initiation of treatment criteria have evolved from a pure end-organ damage-based definition to include risk factors of transformation to frank myeloma. Besides, the mainly cytogenetically defined Multiple Myeloma (MM) risk status, transplant eligibility of patients still serves primarily to allocate patients within a rational treatment algorithm.

While all transplant-eligible MM patients should receive a triplet induction therapy followed by autologous transplantation and, in most cases, lenalidomide maintenance, other therapeutic elements (e. g., other maintenance strategies, consolidation, tandem transplantation,..) have to be decided on an individualized appraisal of risk and toxicities. Standard-risk patients should never be undertreated, as they derive the highest relative benefit from using the best available registered therapies. However, high-risk patients should be preferentially treated inside clinical trials testing additive innovative treatments, as the improvement in the prognosis of this group of patients by standard therapies has been underwhelming. Furthermore, the evaluation process of non-transplant-eligible patients should always comprise an evaluation of performance status, frailty, and comorbidities (e. g., a comprehensive geriatric assessment) to facilitate the allocation of individualized therapies.

## Introduction

Despite the fact that the treatment of newly diagnosed multiple myeloma has changed dramatically over the past 20 years, myeloma remains an incurable, chronically relapsing disease in most patients. Thus, there is still a major unmet medical need to develop more efficacious and less toxic treatments. 

The recent introduction of multiple new agents in MM therapy, usually combined with one another, older drugs, steroids and/or conventional chemotherapy in doublet, triplet, or even quadruplet fashion, has led to a confusing cornucopia of treatment regimens and sequences. Despite an abundance of (mostly phase II) clinical trials in the field, the definition of unambiguous standards of care seems an impossible task in the absence of direct randomized comparisons between a lot (and mostly the most recent) of these options, different availabilities of drugs in different health care systems, and the underrepresentation of important subgroups of patients in clinical trials (e. g., frail, elderly, and comorbid patients). Any attempt (like this one) to summarize standards of treatment in the fast-moving field of MM therapy will thus unavoidably always carry a subjective notion based on the individual experiences of the authors.

**Fig. 1:**
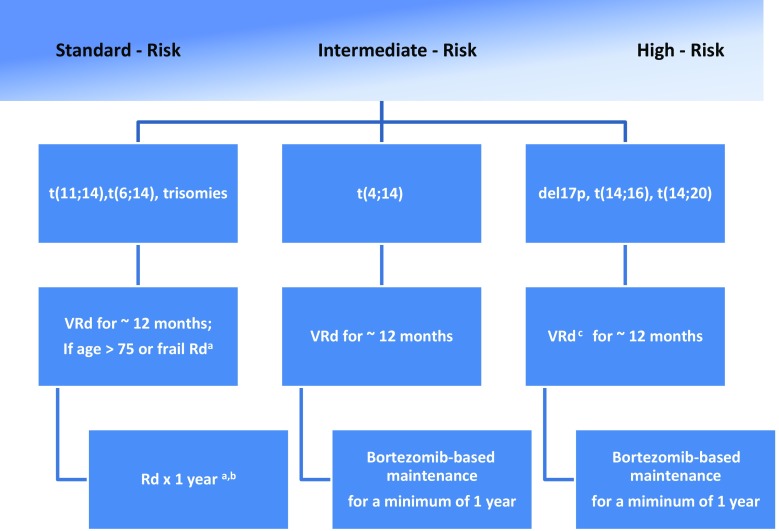
First-line treatment of transplant-ineligible multiple myeloma (*t* translocation, *del* deletion, *V* bortezomib, *R* lenalidomide, *d* low dose dexamethasone). ^a^In patients treated initially with Rd, continuing treatment until progression is an option for patients responding well with low toxicity. ^b^Dexametahsone is usually discontinued after 1 year. ^c^Clinical trial strongly recommended as first option. [1]

**Fig. 2:**
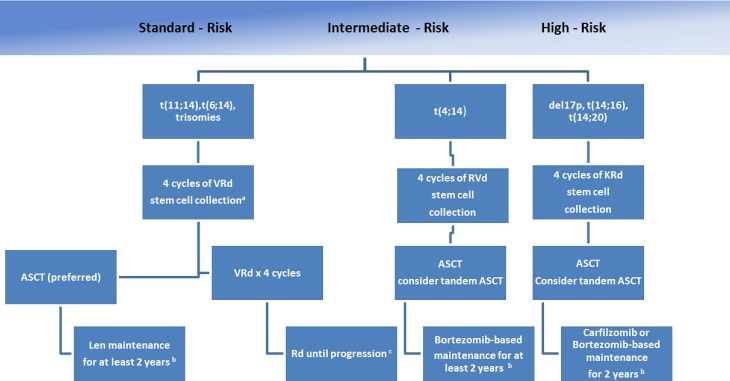
First-line treatment of transplant-eligible multiple myeloma (*t* translocation, *del* deletion, *V* bortezomib, *R* lenalidomide, *d* low dose dexamethasone, *K* carfilzomib, *ASCT* autologous stem cell transplantation, *Len* lenalidomide). ^a^If age >65 years or >4 cycles of VRd, consider mobilization with G‑CSF plus cyclophosphamide or plerixafor. ^b^Duration based on tolerance, consider risks and benefits for treatment beyond 2 years. ^c^Continuing Rd for patients responding to Rd and with low toxicities. [1]

Figures 1 and 2 summarize our treatment policies adapted from the mSMART guidelines [1]. mSMART represents the results of a regularly updated consensus process of the myeloma teams at the Mayo clinic, not a systematic review. It takes into account the results of published clinical trials, as well as the individual experiences of all involved investigators. Thus, mSMART appraises both objective, as well as subjective criteria and not all recommendations are based on published full papers alone. Of course, these recommendations have to be read accordingly and interpreted carefully and critically.

## When to treat

Evaluating the right time to initiate treatment of myeloma patients, clinicians should clearly define the stage of the disease, as well as biologic risk factors and an individual patient’s disposition. The International Myeloma Working Group (IMWG) traditionally recommended starting treatment if a newly diagnosed MM (NDMM) patient presented with symptomatic disease [2] as defined by end-organ damage using the so-called CRAB criteria: hyper*C*alcemia, *R*enal insufficiency, *An*emia, and *B*one disease, and/or the presence of any other clinically significant organ dysfunctions such as an increased occurrence of infections, the development of paraprotein-related polyneuropathy, etc. The definition of symptomatic myeloma has recently been revised by the International Myeloma Working Group [3], Table 1, including the presence of >60% plasma cells, an sFLC ratio >100, or more than one focal bone lesion greater than 0.5 mm on a diffusion-weighted whole-body MRI. Patients achieving these criteria should be treated due to their very high risk (80% over a two-year period) of transformation to overt myeloma. This recommendation has not remained unopposed, as this treatment policy logically results in at least a 20% overtreatment rate. Careful clinical evaluation and a thorough, open-minded discussion with the patient considering age, comorbidities, performance status, and individual life concepts is thus mandatory in any treatment decision in MM, especially if based on SLIM-CRAB criteria alone.

**Table 1:** IMWG Criteria for treatment initiation in Multiple Myeloma [3]

**(A) Classic CRAB criteria**	
Evidence of end-organ damage that can be attributed to the underlying plasma cell proliferative disorder, specifically	Hypercalcemia: serum calcium > 0.25 mmol/L (>1 mg/dL) higher than the upper limit of normal or >2.75 mmol/L (>11 mg/dL)
	Renal insufficiency: creatinine clearance <40 mL per minute or serum creatinine > 177 µmol/L (>2 mg/dL)
	Anemia: hemoglobin value of >20 g/L below the lowest limit of normal, or a hemoglobin value < 100 g/L
	Bone lesions: one or more osteolytic lesion on skeletal radiography, CT, or PET/CT. If bone marrow has <10% clonal plasma cells, more than one bone lesion is required to distinguish from solitary plasmacytoma with minimal marrow involvement
**(B) “SLIM-CRAB” criteria defining “early myeloma” (in the absence of A criteria)**	
Any one or more of the following biomarkers of malignancy	60% or greater clonal plasma cells on bone marrow examination
	Serum involved/uninvolved free light chain ratio of 100 or greater, provided the absolute level of the involved light chain is at least 100 mg/L (a patient’s “involved” free light chain—either kappa or lambda—is the one that is above the normal reference range; the “uninvolved” free light chain is the one that is typically in, or below, the normal range)
	More than one focal lesion on MRI that is at least 5 mm or greater in size

## Treatment for transplant-eligible patients

Response to treatment and survival of newly diagnosed multiple myeloma (NDMM) is heterogeneous, with median overall survival (OS) ranging from less than 2 to >10 years [4]. MM is characterized by chromosomal instability, and cytogenetic aberrations (CA; e. g., deletion 17p) have a major impact on prognosis [3, 5–7].

Recently it has been elegantly demonstrated that for patients with standard-risk features defined by fluorescence in situ hybridization (SR-FISH) undergoing early autologous stem cell transplantation (ASCT), the median overall survival (OS) nowadays approaches 10 years [8]. For patients with high-risk features, including the immunoglobulin heavy chain gene translocations: t(4;14); t(14;16) and copy number changes such 1q gains, 1p losses, and, most of all, 17p deletions, outcomes have also been improved by modern therapies, but much less impressively, approaching an OS of around 4 years [4]. Therefore, the International Myeloma Working Group recommends defining the patients risk status using a combination of FISH results, LDH levels, gene-expression profiles (GEP70), and the International Staging System (ISS) stage for risk stratification in this patient population [4].

## Standard risk—newly diagnosed multiple myeloma

High-dose therapy (HDT) with autologous stem cell transplantation (ASCT) after triplet induction (e. g., VCd [9]; VTd; [10] , VRd [11], KRd [12]) is considered the standard of care for transplant-eligible patients with newly diagnosed MM. Not all of these triplets have been registered in Europe (e. g., VRd and KRd) and their availability is quite diverse, depending on local factors. All patients with nearly diagnosed multiple myeloma who are considered candidates for ASCT (up to 65 years of age in most European centers and up to 75 years of age in the US) should receive 4 to 6 cycles of induction therapy to reduce tumor burden and improve the symptoms.

Nowadays, a triplet induction therapy is considered standard of care, due to benefits of higher overall response rates and improved depth of remission compared to doublet therapy regimes across all risk groups, and is probably most important for the high-risk patient population such as those with 17p deletion and/or t(4;14) translocations [1, 13].

Stem cell collection should always be performed with the goal to collect stem cells for at least two transplantations, as ASCT may comprise a valuable salvage option later. High-dose melphalan is nearly universally used as a conditioning regime globally.

Ample data prove the superiority of early ASCT compared to extended novel agent-based combination therapy with deferred ASCT at progression, as illustrated by the interim results of the IFM/DFCI trial reported at ASH 2016 [14] with respect to PFS. On the other hand, in the most recent trials, although with short follow-up, no OS benefit for ASCT could be shown on.

In patients not achieving at least a very good partial response (VGPR), several strategies are under discussion: tandem ASCT, alone or in combination with consolidation and/or maintenance therapies [15]. For patients with standard-risk cytogenetics who achieved a very good partial remission (VGPR) or better, the data for a benefit of consolidation therapy are less clear as yet and should be used in the framework of prospective clinical trials only [12].

Lenalidomide-based maintenance after ASCT has shown clear superiority with respect to PFS and OS vs. no maintenance in two of three big fully published randomized trials (e.g., [16]) and, most of all, in a subsequent meta-analyses [17]. Data on a fourth trial were shown at ASH 2016 and 2017 [18], confirming both the PFS data of the other three [19], as well as an OS benefit in TE patients. Furthermore, this trial (MRC Myeloma XI) confirmed, in contrast to other trials, a benefit of lenalidomide maintenance in high-risk TE patients (defined by cytogenetics and/or ISS and LDH levels). All these facts contributed to the approval of lenalidomide-based maintenance by both FDA and EMA. Therefore, most experts nowadays recommend the use of lenalidomide maintenance post ASCT in all patients. Whether high-risk patients would profit from additive maintenance strategies (applying antibodies or oral proteasome inhibitors, using tandem or auto-/allotransplant approaches) besides the use of lenalidomide is currently under investigation in multiple clinical trials, while none of these options have been registered in these indications. Nevertheless, despite a lack of data from clinical trials, to consider strategies as adding a proteasome inhibitor or an anti-myeloma monoclonal antibody in this clinical setting on an individualized basis was proposed at the IMW meeting 2017 in New Delhi by N. Raje in her talk on a new IMWG guideline on HRMM (not yet published).

## High risk—newly diagnosed multiple myeloma

The management of newly diagnosed high-risk myeloma patients is still a formidable challenge [4, 7]. In transplant-eligible patients, the hallmark of first-line treatment is high-dose therapy and ASCT combined with novel agents. This strategy has significantly improved PFS and OS [4].

The IMWG consensus statement for treatment of HR-NMM recently published by Sonneveld et al. guides clinicians with respect to treatment allocation in these difficult-to-treat patients [4]. For transplant-eligible HR-MM it recommends to use, as a minimum, the sequence of a triplet induction therapy, including proteasome inhibitors and an immunomodulatory drug as well as a corticosteroid.

Summarizing multiple clinical trials, the consensus lists several points to be kept in mind with respect to treatment decision in HR-NDMM:

*Thalidomide* does not abrogate the adverse effects of IgH translocations: t(4;14), t(14;16), t(14;20), as well as del17p, and amp1q cytogenetic abnormalities (CA) in transplant eligible (TE) patients [4].

*Bortezomib* partly overcomes the adverse effect of t(4;14) and possibly del 17p on CR, PFS, and OS. There is no effect in t(4;14) combined with del17p in TE patients.

*Lenalidomide* partly improves the adverse effect of t(4;14) and del17p on PFS, but not with respect to OS, in TE patients.

*Combining a proteasome inhibitor with lenalidomide and dexamethasone* greatly reduces the adverse effect of t(4;14) and/or del17p on PFS in NDMM [4].

*Carfilzomib with lenalidomide and dexamethasone* seems most effective in patients with HR cytogenetics [20].

*Just updated data on the effect of tandem HDT/ASCT* combined with bortezomib-based induction show an improved PFS and also OS [21] in patients with t(4; 14) or del17p, as well in those with both abnormalities. Although the American StaMINA [22] trial could not corroborate these findings, HDT plus double ASCT is recommended for patients with HR cytogenetics [23].

*Allogenic stem cell transplantation* in young patients with HR NMM is highly controversial due to limited data from randomized trials, severe toxicities, an excessive early death rate, and multiple new alternatives. The IMWG consensus statement with respect to allo-SCT is that allogeneic SCT or tandem auto-allo-SCT may improve PFS in patients with t(4;14) or 17p deletion, with results being better in earlier stages of the disease. Yet novel treatments continuously challenge the role of allo-SCT, which should ideally be restricted to use in clinical trials only [4].

Whenever feasible, high-risk patients should be treated inside clinical trials testing additive innovative treatments.

## Treatment for transplant-ineligible patients

Criteria to define transplant ineligibility are not well defined and may vary with different health care settings, but most clinicians would agree that using structured measurements of performance status or a frailty index, rather than pure age, are better instruments to determine the best treatment approach for older patients with myeloma [24]. The International Myeloma Working Group has created a geriatric online assessment [25] tool based on age, comorbidities, cognitive capacity, and physical capabilities and identified three groups: fit, intermediate, and frail patients. The use of this or other assessment tools has been shown to reliably predict survival and toxicities. This assessment thus helps clinicians to identify the patients for adequate treatments and protect them from both unnecessary toxicity as well as under treatment. Guidelines on age- and comorbidity-based tailored therapy have been published [26] by the European Myeloma network (EMN).

The usefulness of such an approach is further illustrated by the results of the UPFRONT [27] trial, a community-based phase IIIB US trial that compared three bortezomib-based regimes (VD, VTD, VMP) in a quite elderly (median age 73 years) “realistic” NTE population and found no advantage for the triplets vs. doublets combinations. Thus, higher treatment intensity does not necessarily translate to better testament efficiency in elderly populations.

The goal of upfront treatment is to achieve the deepest response possible, as outcomes correlate with the depth of response, while protecting the patient. These findings further emphasize the need for active and effective therapies for older patients and, at same time, meet the challenge of balancing the side effects and burden of treatment, which are more pronounced in this population.

In this era of new drugs, patients who are not transplant eligible are nevertheless able to achieve deep responses using intelligent combination therapy approaches, including most commonly a proteasome inhibitor, an immunomodulatory drug, and a corticosteroid in toxicity-adapted fashions. For example, there are data from a phase III study suggesting that triple therapy with VRd (bortezomib, lenalidomide, and dexamethasone) is able to achieve an overall response rate (ORR) of up to 82% in NTE-NDMM [28]. This trial showed superior outcomes with VRd compared to a standard doublet therapy with lenalidomide and dexamethasone (Rd) alone. VRd vs. Rd: (median PFS 43 vs 30 months; p = 0.0018), overall survival of 75 versus 64 months. To reduce side effects, VRd was modified in a “VRd lite” regime (bortezomib 1.3 mg/m^2^ body surface on day 1, 4, 8 and 11, lenalidomide 25 mg on days 1–14, and dexamethasone 20 mg on d 1, 2, 4, 5, 8, 9, 10 ,11, to be repeated every 3 weeks). This modified regime showed an excellent tolerability.

Another promising approach to deepen responses and improve efficacy of first-line treatment in NTE patients is the introduction of immunotherapies (e. g., anti-myeloma antibodies) in the front-line setting.

The recently published Alcyone trial [29] investigated the concept of adding daratumumab to a standard VMP regime in a phase III randomized trial and could demonstrate a lower risk of disease progression or death than the same regimen without daratumumab. The 18-month progression-free survival rate was 71.6% (95% confidence interval [CI], 65.5 to 76.8) in the daratumumab group and 50.2% (95% CI, 43.2 to 56.7) in the control group (hazard ratio for disease progression or death 0.50; 95% CI, 0.38 to 0.65; *P* < 0.001). On the other hand, the daratumumab-containing regimen was associated with more grade 3 or 4 infections (23.1% vs. 14.1%).
